# Assessment of the hazard risks on HVDC transmission networks due to lightning strikes and faults

**DOI:** 10.1371/journal.pone.0313229

**Published:** 2024-12-13

**Authors:** Abdelrahman Said, Mohamed Galal, M. A. Abd-Allah

**Affiliations:** 1 Department of Electrical Engineering, Faculty of Engineering at Shoubra, Benha University, Cairo, Egypt; 2 Faculty of Computer Science, Benha National University (BNU), Al Obour, Egypt; Joginpally B R Engineering College, INDIA

## Abstract

HVDC systems with high voltage and direct current are suitable for reducing electricity losses, during transmission over long distances. However, HVDC lines have the highest probability of failure within a system and can be damaged by different faults or lightning strikes. This paper presents a transient circuit model for the practical Egypt–Saudi Arabia link. The transient potential at different points along the line is calculated under different magnitudes and shapes of lightning strokes. "Additionally, the extent of transient overvoltages at the rectifier station due to shifts in lightning strike locations has been estimated. Furthermore, overvoltage distributions are examined caused by lightning strikes on a real ± 500 kV hybrid HVDC TL-Cableare examined. To prevent national disasters and maintain safe national ratios of electrical production, particularly in the case of hybrid HVDC TL-Cable faults, an analysis of different AC/DC faults in the hybrid HVDC TL-Cable is also presented. This study is based on an equivalent electrical model using Power Systems Computer-Aided Design (PSCAD) software. It was discovered that severe voltages appear at the rectifier station when 50 kAlightning stroke with 1.2 μ sec front time, and 350 μ sectail time hits the TL. Moreover, overvoltages are reduced by about 58% in hybrid HVDC TL-Cable. Also, surge arresters at rectifier stations reduce overvoltages by approximately 95%. Ultimately, DC pole-to-ground faults are more serious than other types of faults and can cause massive overcurrent in the system, potentially damaging the rectifier station.

## 1. Introduction

According to expectations, the future will witness a greater reliance on renewable energy sources, including wind farms (both offshore and onshore), solar power stations, and hydroelectric plants [1]. For many nations, a secure, long-term, affordable energy source is the most important thing, and this can be provided by HVDC transmission systems, which are capable of transmitting large amounts of power, especially which is vital for the stability of the power grid. However, when power is transmitted over long distances (e.g., 500 km), issues like reduced power transfer capability, voltage profile variations, and reactive generation may arise [2]. Therefore, HVDC transmission has been suggested as an ideal solution for connecting massive offshore renewable energy resources [3, 4]. Voltage-sourced converters (VSCs) in HVDC systems include line-commutated converters (LCC) and modular multilevel converters (MMC) [5]. The LCC-HVDC system employs thyristor-switching devices that operate in the current switching mode [6, 7]. The thyristor-based LCC-HVDC system consumes reactive inductive power because its conduction is unidirectional without forced blocking capability, hence causing phase displacement between current and voltage [7, 8]. Another HVDC technology, MMC-HVDC, uses an IGBT based VSC with bi-directional current conduction ability and separate control for both real and active power is used. Although many articles tried to cover different aspects of lightning transients in mixed HVDC, several researchers have studied the impact of lightning strikes on composite transmission lines [9–12]. Nevertheless, bipolar lightning strikes occur in multi-flashes, thereby resulting in transient overvoltages due to subsequent strokes coupled with wave interactions within mixed HVDC links [13, 14]. Since HVDC transmission lines rank among components having high failure rates within the system and can suffer from different fault types i.e., dc faults or ac faults commonly pole-to-ground fault being one [15]. Consequently, it is imperative to differentiate between lightning disturbances, lightning-induced faults, and non-lightning faults. Furthermore, it is necessary to differentiate transient faults caused by lightning strikes from ordinary short-circuit faults. Analyzing these fault characteristics is of significant practical importance for ensuring the security of power system operations. Numerous studies have concentrated on the causes of induced overvoltages [16]. Consequently, in order to guarantee the necessary safety for power supply and electromagnetic compatibility, it must be calculated in advance as overvoltage due to lightning strikes. Several variables influence the amount of overvoltage produced when lightning strikes an overhead transmission line. These include peak lightning current, rate-of-rise of that current, tower resistance, and surge impedance [17]. The results of studies concentrate on assessing surge voltages caused by lightning where shielding failure flashover and back-flashover through insulators are considered with different peak values for back flashover and shielding-failure flashover under various peak values of lightning currents. Additionally, they also investigate how changing DC grounding resistance affects potential across towers, leading to either more likely back flashovers or shield failures depending on whether the resistance is increased or decreased [18–20].

Several investigations have been conducted on different aspects affecting performance of DC transmission lines such as location and capacity determination for this type of lines among others geometric parameters used during the design stage, as well as fault types and their locations [21]. Some works done concerning HVDC faults give deeper understanding about fault location methods employed when dealing with these kinds of faults especially around Single-Ended Traveling-Wave Fault Locating method, natural frequency method, fault analysis method [22, 23]. In relation to HVDC line fault diagnosis system faults mechanism is investigated by [24]. The fault equivalent circuit also used for finding minimum fault identification level hence it classifies single-pole short-circuit, double-pole short circuit, and single pole disconnection.

The studies reviewed so far mainly focus on induced components under normal condition without considering transient state effects caused by faults or other disturbances on these components. As mentioned earlier, research work has studied what happens if we change our grounding, which establishes that modeling techniques like the selection of wave shape for lightning stroke current and insulator criterion flashover voltage can cause wide variations in lightning surge overvoltages of DC lines. However it should be noted that transient states like lightning or faults have great influence on induced components although many more researches need to be carried out regarding severity assessment for transient overvoltage in HVDC transmission systems.

This paper presents a comprehensive transient circuit model for the Egypt-Saudi Arabia HVDC link, rated at ±500 kV and 3000 MW, with 500 rectifier transformers on both ends. A thorough assessment analyzes the transient voltage at the rectifier station for the HVDC transmission line and the hybrid HVDC TL-Cable line. The transient overvoltage at various locations along the HVDC line during direct lightning strikes is studied using PSCAD. Additionally, a surge arrester mitigation technique is proposed to protect the rectifier stations. Furthermore, the paper investigates the impact of AC/DC faults in the hybrid HVDC TL-Cable line.

The significant contributions of this study are as follows:

- Evaluation of induced overvoltages in the HVDC line and hybrid HVDC TL–cable.
- Investigation of the influence of direct lightning strokes with different magnitudes, wave shapes, and AC/DC faults.
- Examination of the relationships between these scenarios considering their locations relative to the rectifier station.

The paper has the following structure: In section 2, we detail system model, with an emphasis on ±500 kV hybrid HVDC TL-Cable line connecting Egypt and Saudi Arabia. Section 3 shows results and discussion, and Section 4 provides an overview of this work.

## 2. HVDC system description and modelling

**Fig 1:**
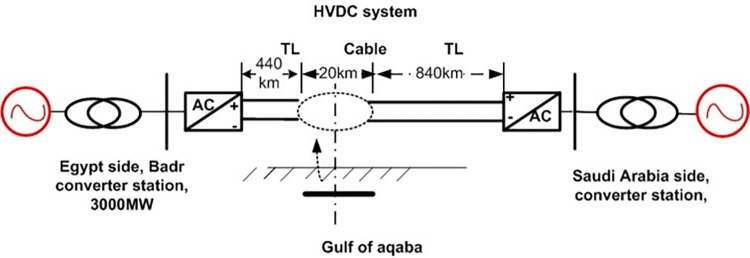
HVDC system.

A rigorous approach to modeling and simulation was employed to analyze transients in various HVDC systems achieving the comprehensiveness and precision required for this paper. The model for the practical Egypt-Saudi Arabia link is a ±500 kV, 3000 MW system with 500 rectifier transformers on each side, as illustrated in Fig 1. The LCC HVDC link extends from Badr City in Egypt to Madinah El Munawara in Saudi Arabia, with the transformers integrated into the grid.

### 2.1 HVDC TL modeling

The Frequency Dependent (Phase) Model is used in order to simulate transmission lines in PSCAD. The phase surge impedance is determined by the inductance and capacitance of conductor or shield wire as indicated in the equation below [25]:

$$z=\surd \frac{L}{C}$$ (1)

Where

$$L=2\times 10^{-7}ln(\frac{Dm}{rB}+\frac{1}{4n})$$ (2)

And

$$C=\frac{2\pi \varepsilon_{{}^{\circ}}}{ln\frac{Dm}{rB}}$$ (3)

Where*Z*, Surge impedance; L, Inductance pulength, Capacitancepu length; *Dm*, Geometric Mean Distance; *rB*, Geometric Mean Radius.

### 2.2 HVDC TL tower modelling

**Fig 2:**
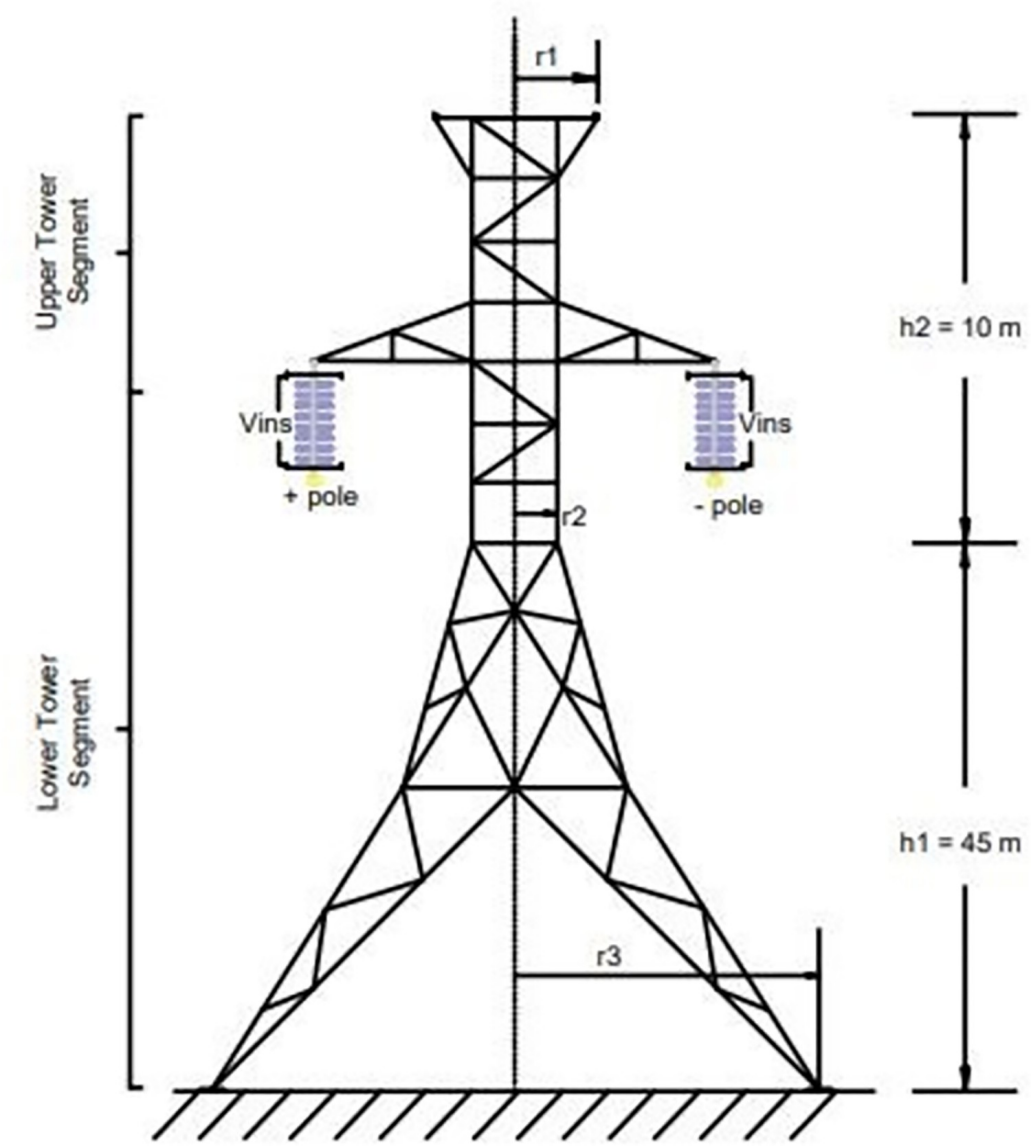
Typical HVDC tower.

The geometry and tower type used in this study are depicted in Fig 2. The influence of tower geometry and tower footing resistance is incorporated into the fast-front transient tower models. In PSCAD, the Frequency Dependent (Phase) Model is used to represent the tower body.

The tower has a wave propagation speed that is 85% of the speed of light [26]. Eq (4) represents the characteristic impedance of the waisted tower structure shown in Fig 2 [27].


$$Z_{T}=60\;ln\{\cot(\frac{1}{2}\tan^{-1}T)\}$$ (4)

where,

T intermediate value $\to \{r_{1}h_{2}+r_{2}(h_{1}+h_{2})+r_{3}h_{1}\}/{\left(h_{1}+h_{2}\right)}^{2}$

*h*_1_ height from base to waist,

*h*_2_ height from waist to top.

*r*_1_ radius of the tower at the top,

*r*_2_ radius of the tower at the waist,

*r*_3_ radius of the tower at the base.

### 2.3. Tower footing resistance

In general, there are two ways to model a tower’s footing resistance. The resistance can be either constant, with a typical value or variable, depending on the magnitude of soil ionization or current [28]. In this paper,the tower footing resistance is modeled as a constant value of 100Ω, and soil ionization is accounted for.

### 2.4 Cable line modelling

**Fig 3:**
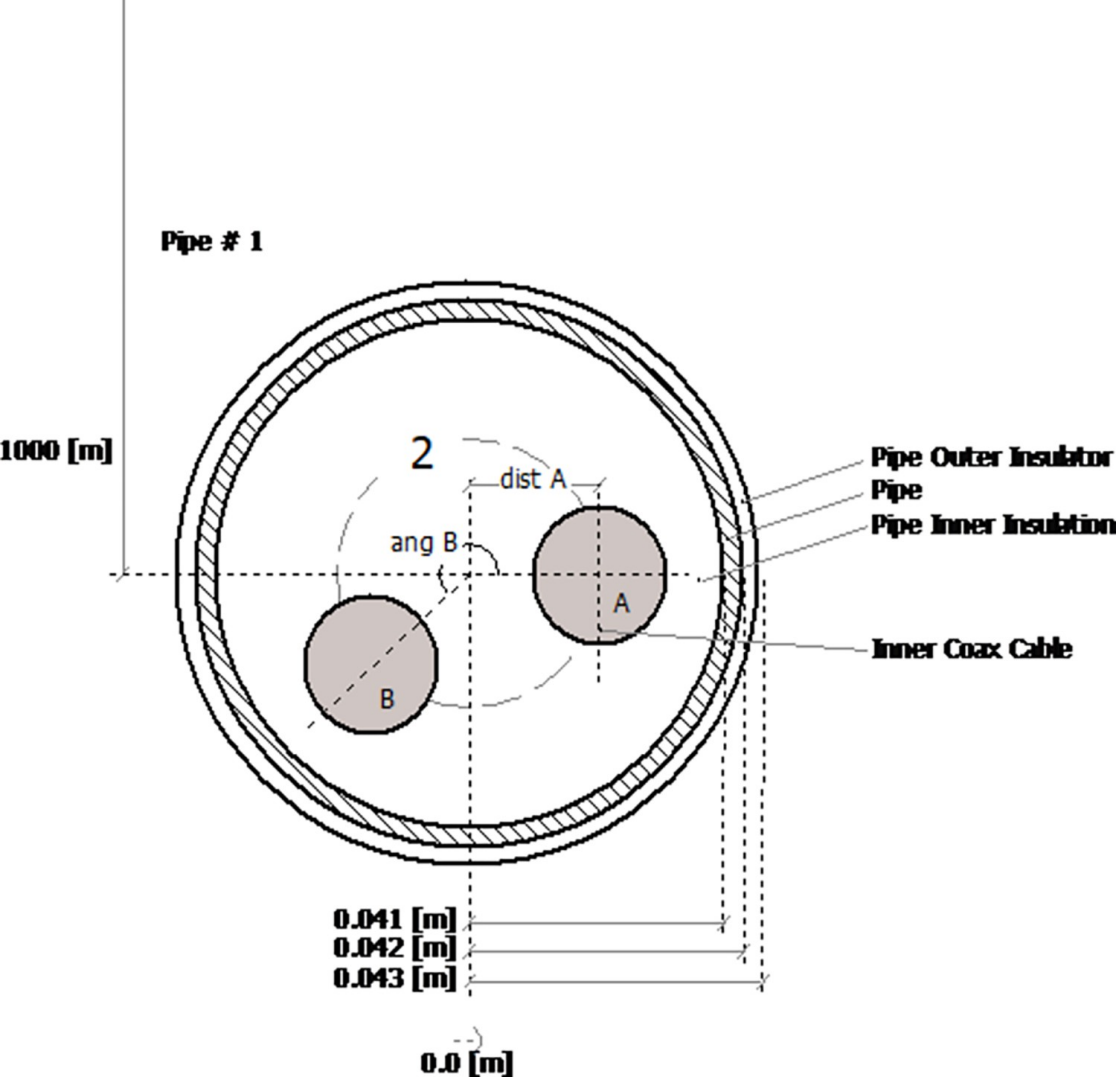
Typical HVDC submarine cable.

The diagram in Fig 3. [29] shows the ± 500 kV submarine cable placement of the bipolar system. The cable path will cross the Gulf of Aqaba, covering a route approximately 20 km in length, with deep-water installation at a depth of about a thousand meters

### 2.5 The lightning model

Lightning sources are modeled as current sources. In this paper, the surge current value will vary between 20 kA and 50 KA when a voltage impulse stroke hits the tower conductor [30].


i(t)=I°(tτ1)2[(tτ1)2+1]e−t/τ2 (5)

Where *I*_°_ represents the maximum value of current, and *τ*_1_, *τ*_2_ are time periods during which current rises or falls.

## 3. Results and discussion

**Fig 4:**
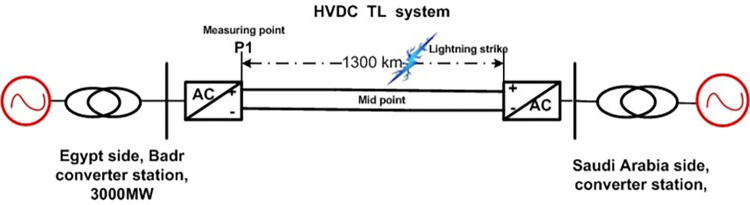
HVDC system model.

Integrating all equivalent circuit models described in previous sections makes it possible to create a PSCAD HVDC model for the transmission line. A lightning strike strikes the transmission line at its middle point, as illustrated in Fig 4.

### 3.1 HVDC TL scenario

In this section, the influential factor on an induced voltage at the rectifier station (P1) is introduced.

#### 3.1.1 The effect of the peak value

**Fig 5:**
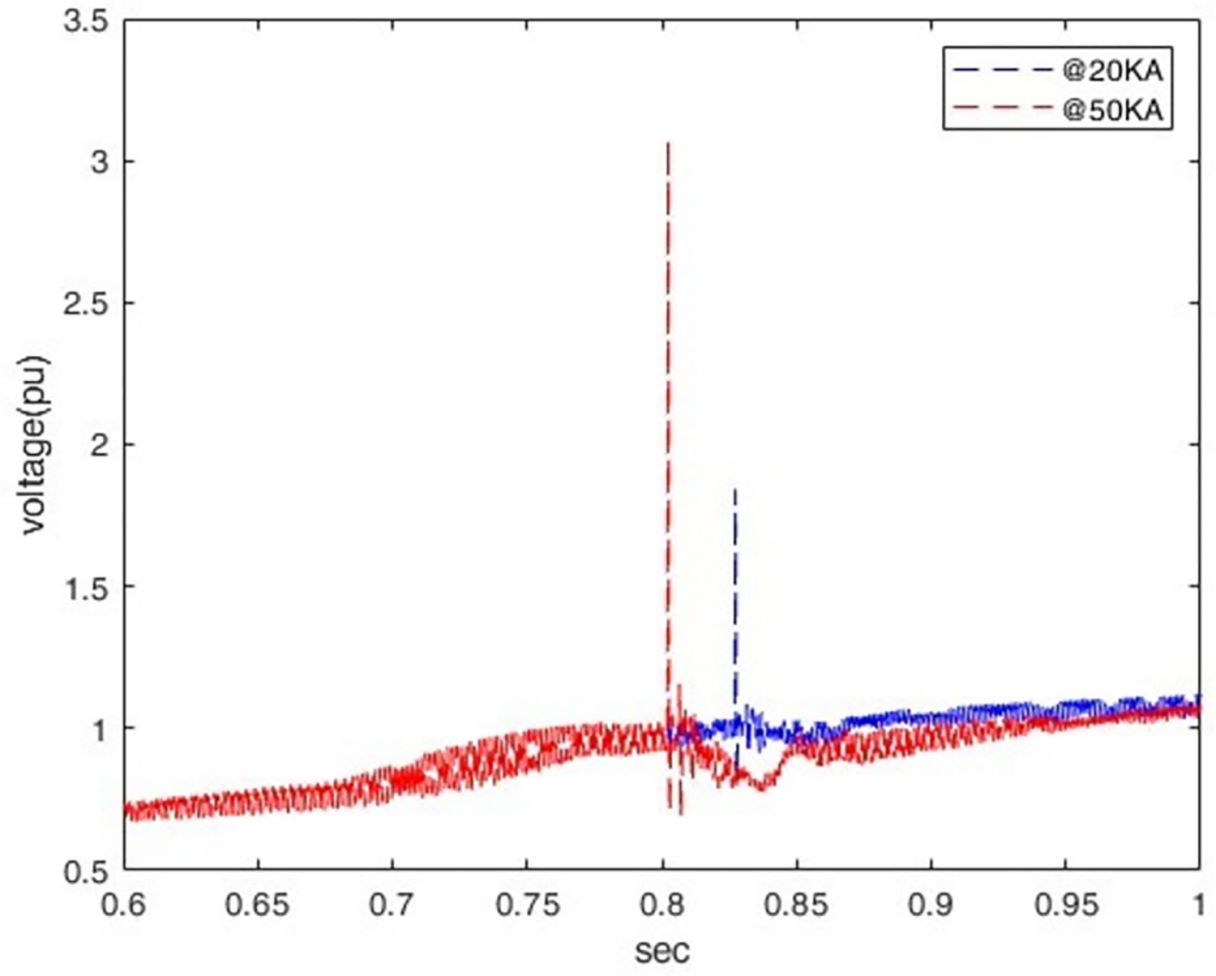
Induced overvoltage at P1.

When a lightning strike occurs in the middle of the transmission line with different peak currents (20 kA, 50 kA) and fixed front and tail times (1.2/50 μs), there is an increase in the peak transient potential and transient overvoltage rate at P1. As shown in Fig 5, an increase in peak lightning current from 20 kA to 30 kA causes the peak transient voltage at P1 (rectifier station) to increase from 1.8 pu to 3.07 pu.

#### 3.1.2. Front time and tail time characteristics effect

**Fig 6:**
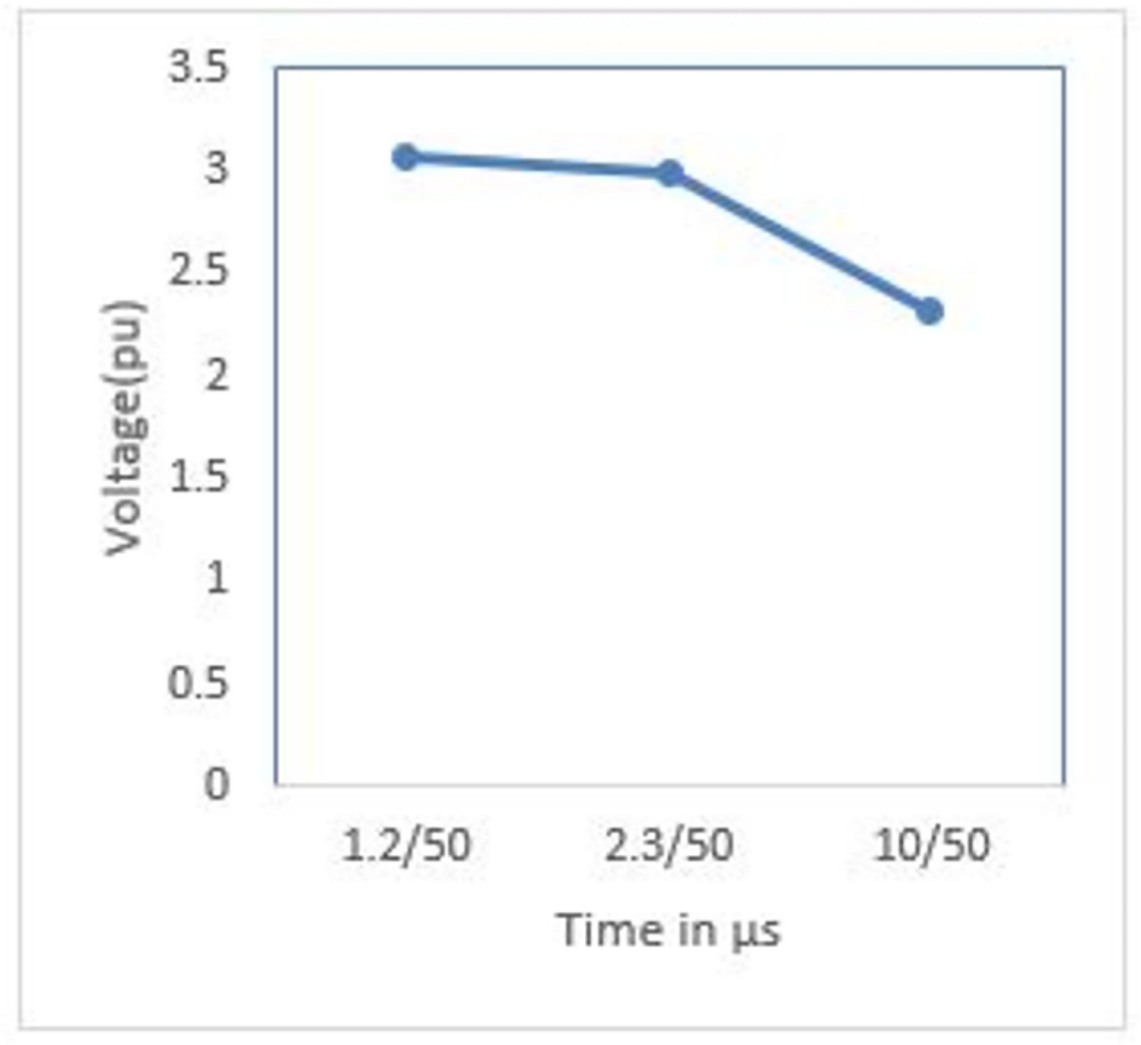
P1 induced voltage under different front time.

**Fig 7:**
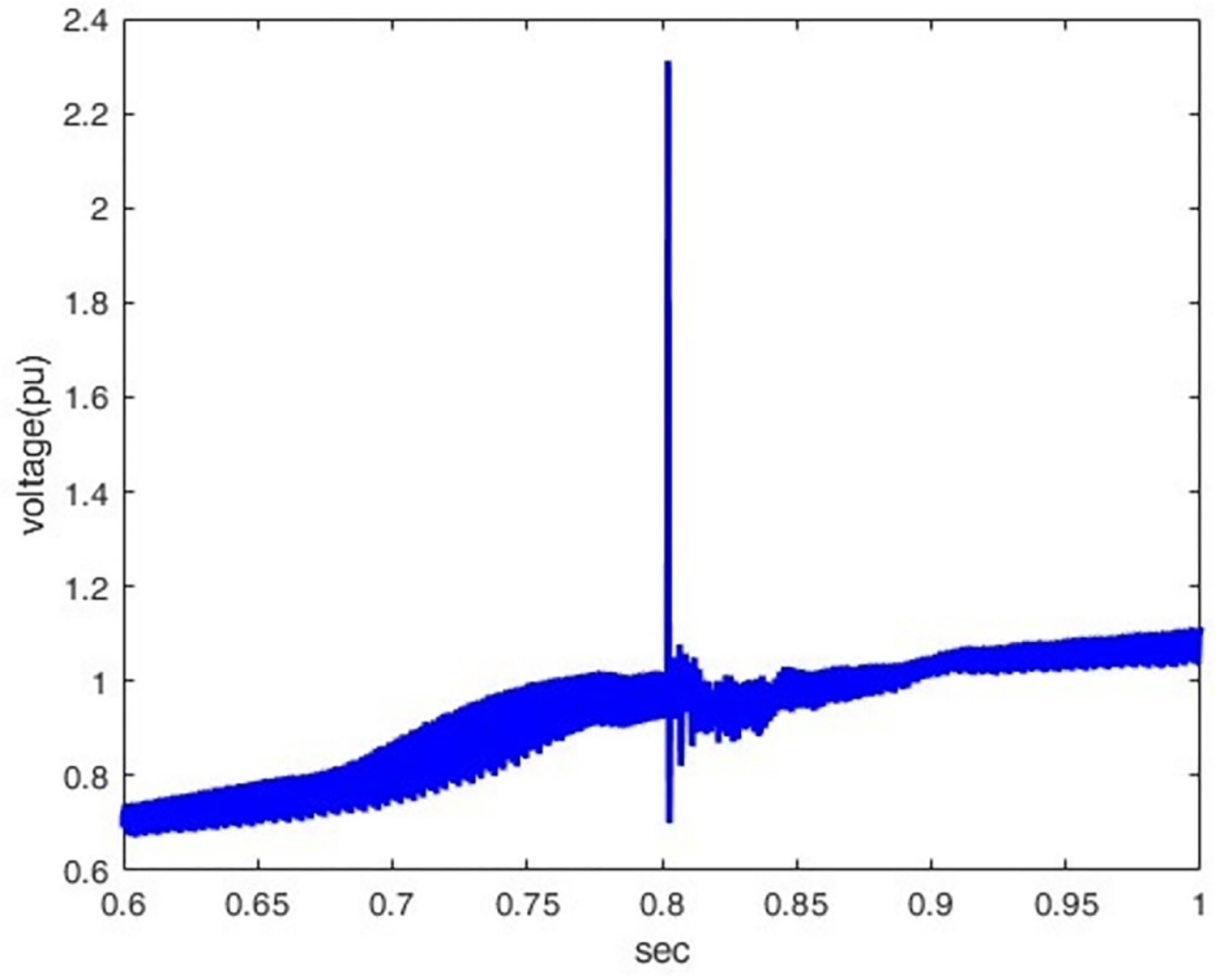
Voltage waveform at 50 KA(10/50) μs.

The study investigates this under a constant lightning current of 50 kA. It is shown in Figs 6 and 7 that the front time increases from 1.2 μs to 10 μs, causing the peak transient voltage at P1 to drop from 3.07 pu to 2.31 pu. Since wave front time is much shorter than tail time, this occurs when we decrease wave front time which in turn increases the high frequency component thereby increasing di/dt value and leading to higher peak transient potential value.

**Fig 8:**
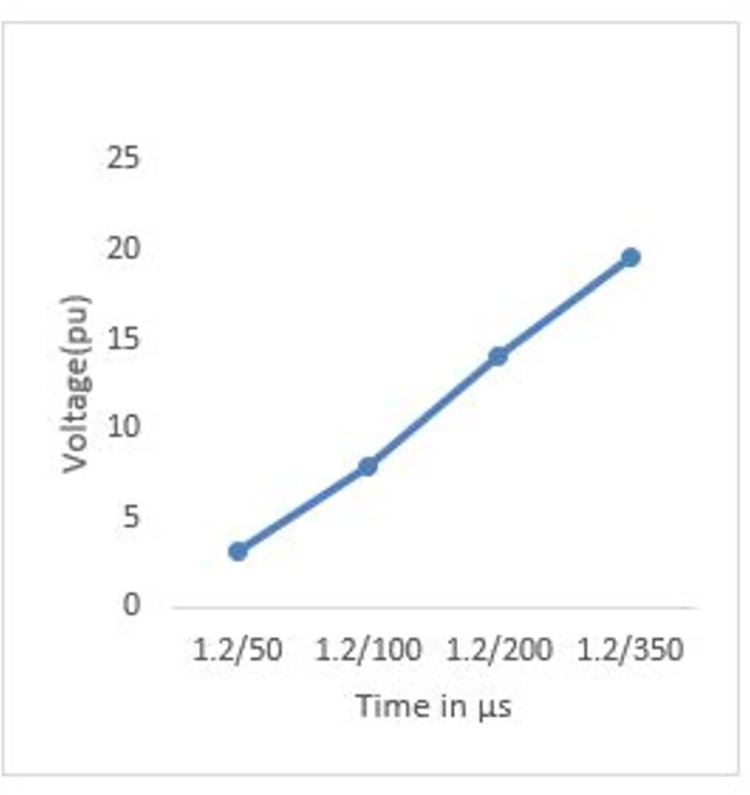
P1 induced voltage under different tail time.

**Fig 9:**
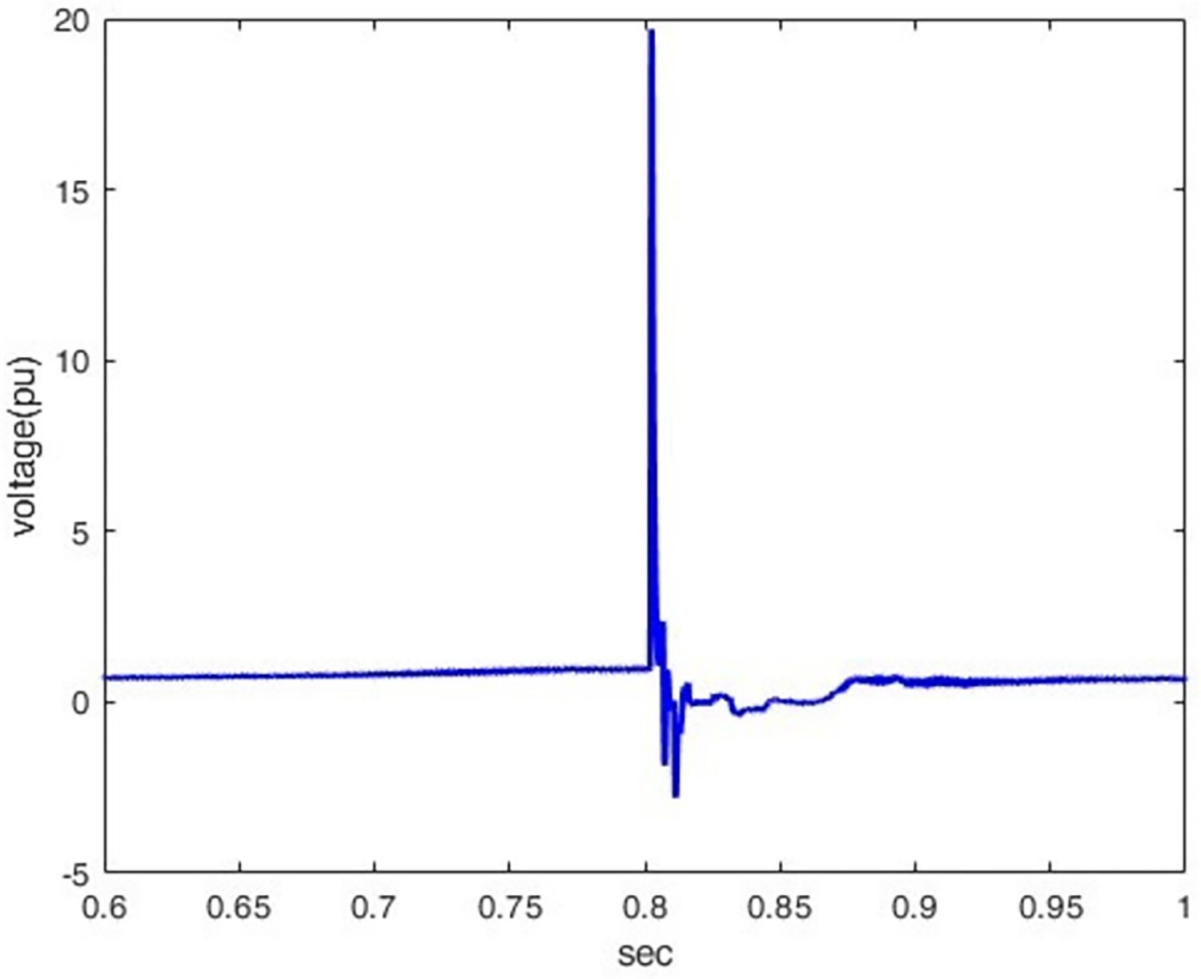
Voltage waveform at 50 KA(1.2/350).

The voltage increases when the wave tail time is extended because it spends more time decaying to its half value after peaking; hence, as shown in Figs 8 and 9, the peak transient potential rises from 3.07 pu to 19.68 pu when the wave tail time increases from 50 μs to 350 μs, thus allowing more accumulated propagated waves which raise the voltage.

### 3.2. Hybrid HVDC TL–cable scenario

This section introduces the behaviour of hybrid HVDC TL–cable under lightning strikes and faults.

#### 3.2.1. Lightning strikes study

**Fig 10:**
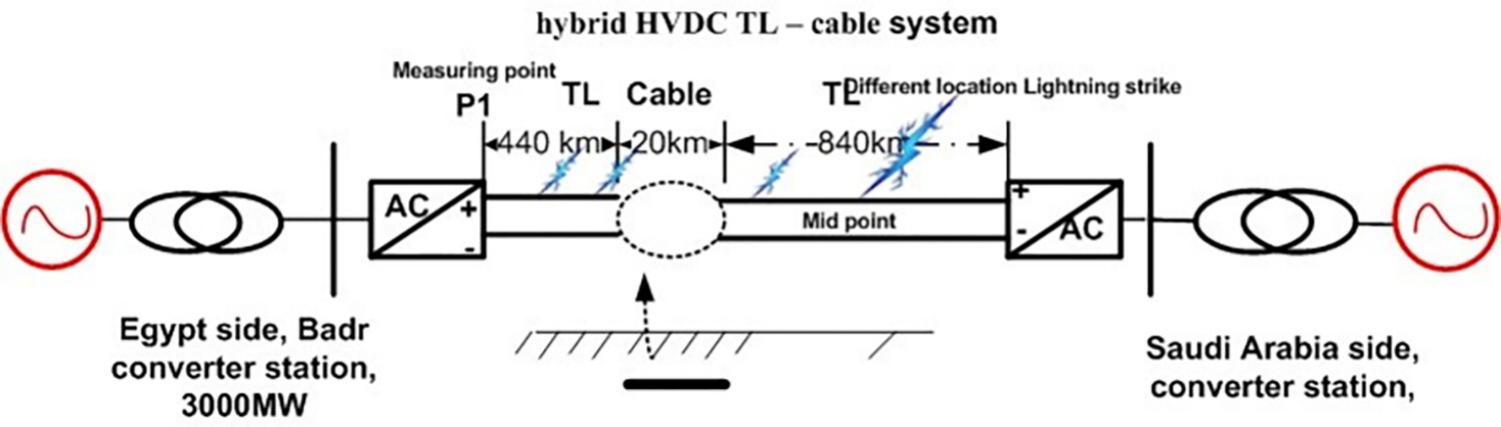
Hybrid HVDC TL–cable configuration.

*3*.*2*.*1*.*1*. *Lightning overvoltage behaviour comparison under two scenarios*. This section simulates lightning for hybrid HVDC TL–cable, which submarine cable route crosses the Gulf of Aqaba with a length of approximately 20 km, as shown in Fig 10. Induced voltage at the rectifier station (P1) was determined when a lightning strike (50 kA-1.2/350 μs) hits the mid-span in the case of HVDC TL and hybrid HVDC TL-cable.

**Fig 11:**
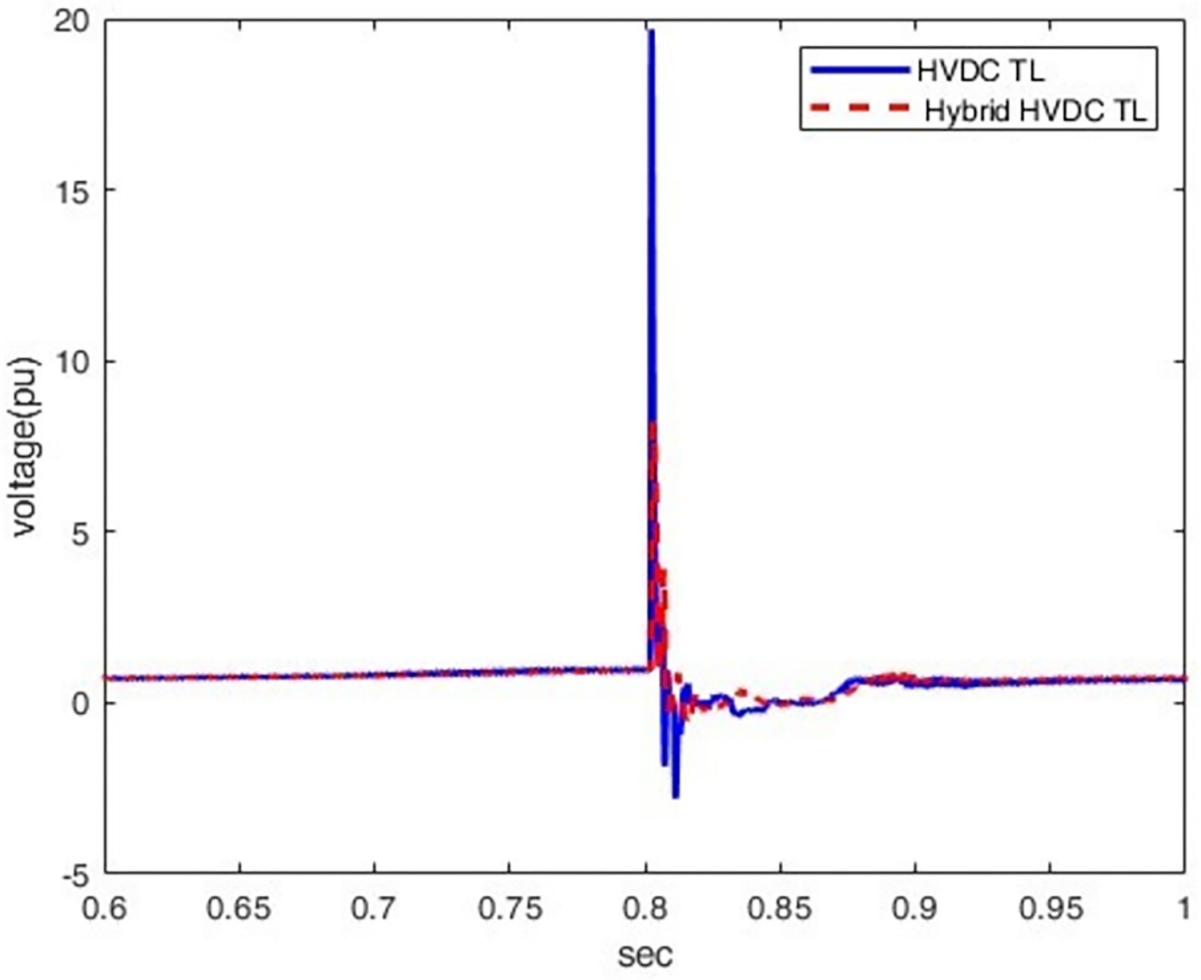
Overvoltage at P1 in the case ofHybrid HVDC TL–cable configurationandHVDC TL configuration.

The results show that the voltage at the rectifier station reaches 8.2 pu. in the case of hybrid HVDC TL–cable (i.e., a reduction of about 58% compared to the HVDC TL configuration), as shown in Fig 11.

**Fig 12:**
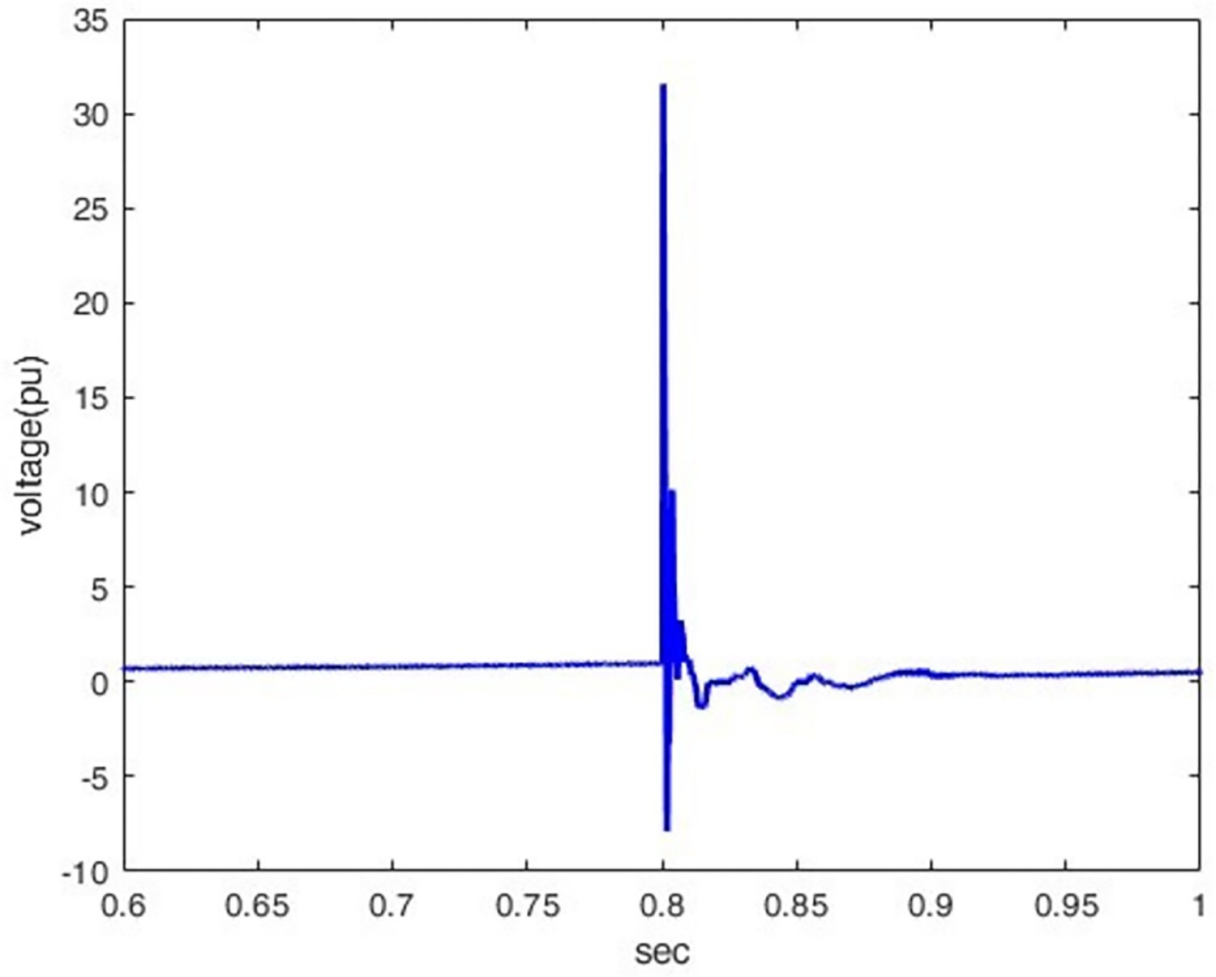
P1 induced voltage from a lightning strike at various distances from the rectifier station: 100 km.

**Fig 13:**
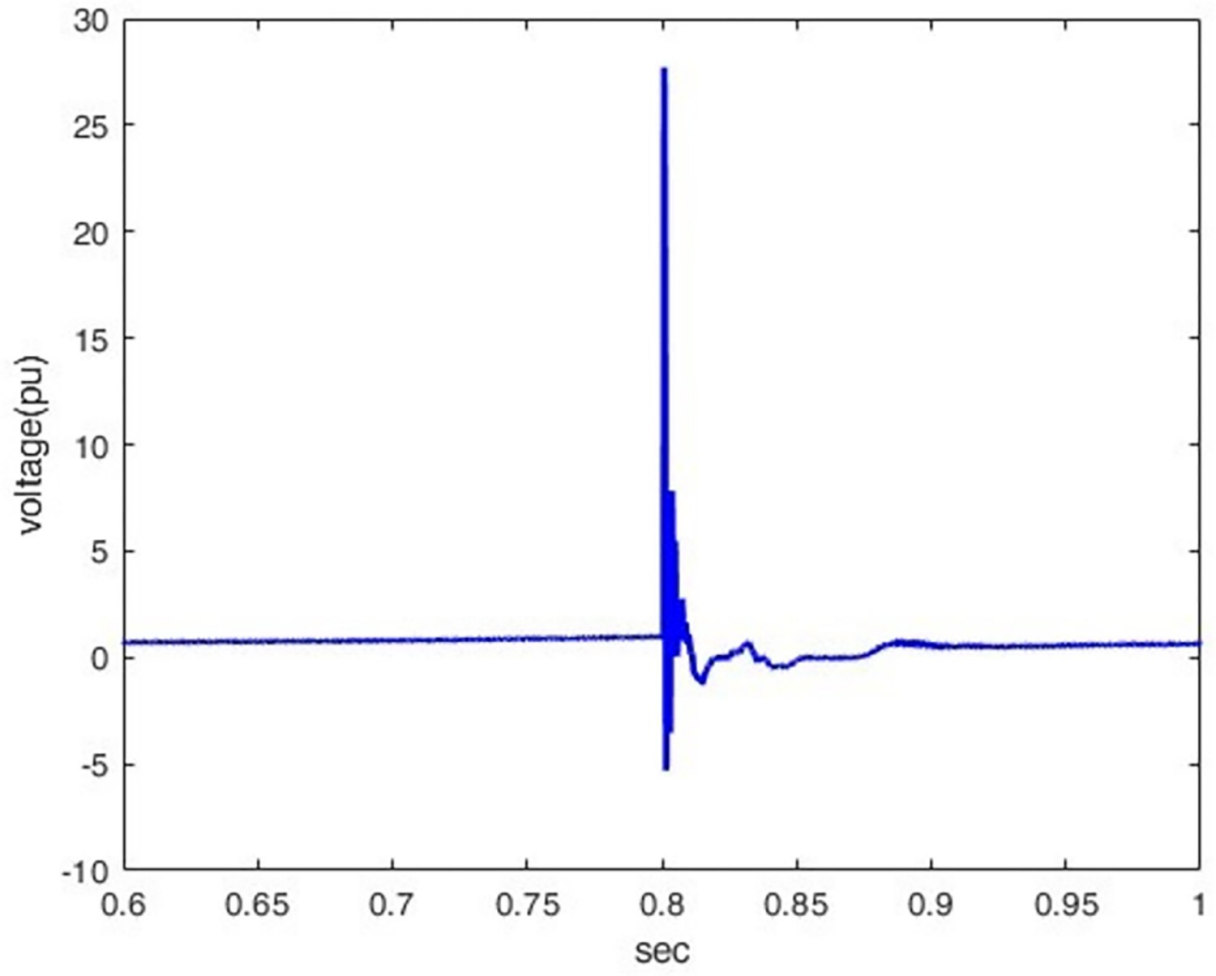
P1 induced voltage from a lightning strike at various distances from the rectifier station: 200 km.

**Fig 14:**
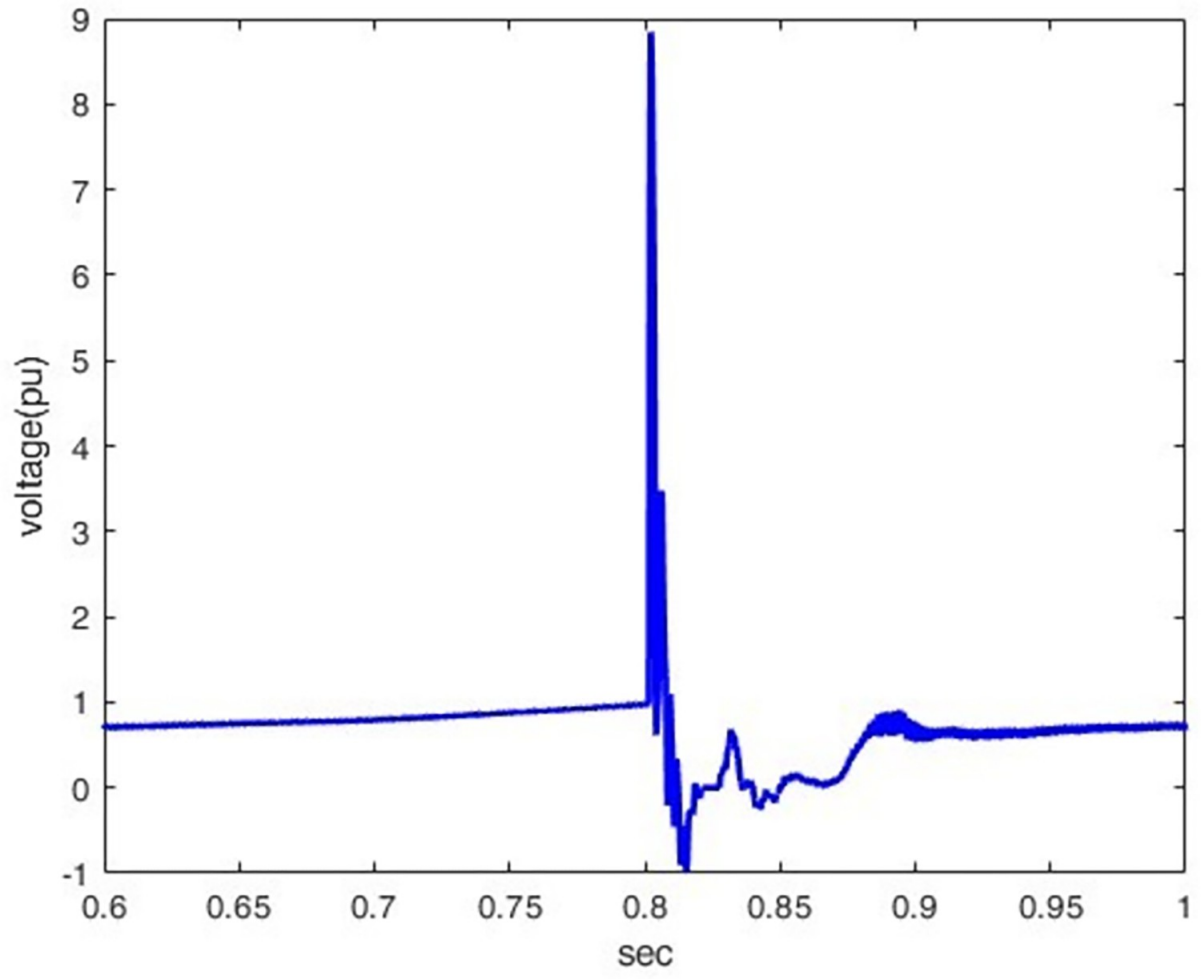
P1 induced voltage from a lightning strike at various distances from the rectifier station: 400 km.

**Fig 15:**
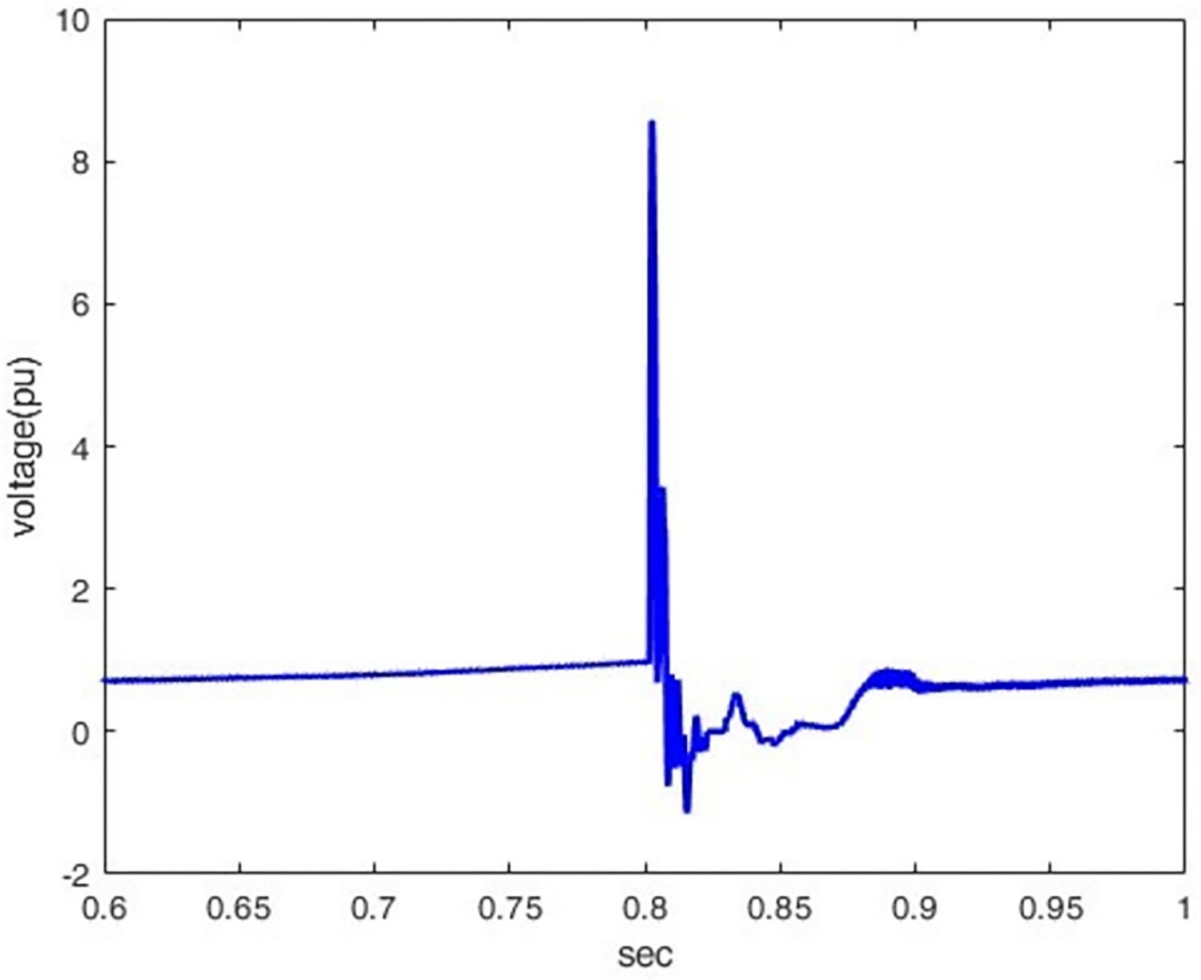
P1 induced voltage from a lightning strike at various distances from the rectifier station: 500 km.

*3*.*2*.*1*.*2*. *Lightning different location effect*. The overvoltage at P1 was determined when lightning stroke (50 kA-1.2/350 μs) hit the line at different locations (100km, 200km, 400km, and 500km) away from P1. Figs 12–15 shows that the nearest lightning to the rectifier station has the highest induced overvoltage, reaching 31.5pu.

**Fig 16:**
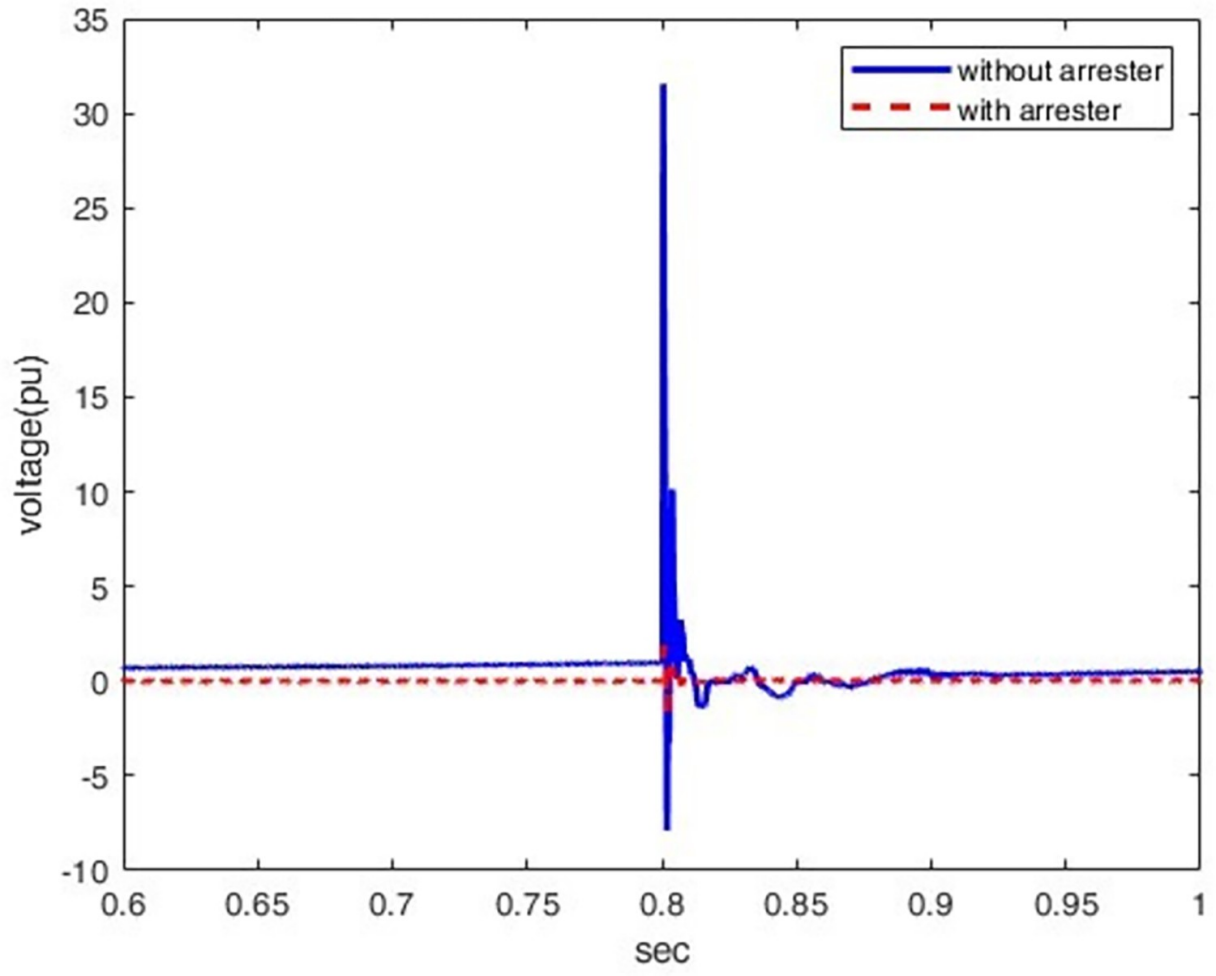
Overvoltage at P1 with and without surge arrester.

*3*.*2*.*1*.*3*. *Surge arrester as mitigation technique at rectifier station*. The lightning stroke creates a very high transient potential at sensitive points where it is observed. At these transients, a significant amount of energy can damage equipment at the rectifier station. Thus, there is a need to lower these large potentials at the rectifier station terminals and prevent them from being damaged by employing a surge arrestor as a shielding method. Fig 16 shows what happens if an arrester is installed to reduce induced overvoltage at P1. According to Fig 16, the induced voltage drops from 31.5 pu down to 1.5 pu, i.e., by 95% mitigating the effect of induced overvoltage at P1.

#### 3.2.2 Faults study

**Fig 17:**
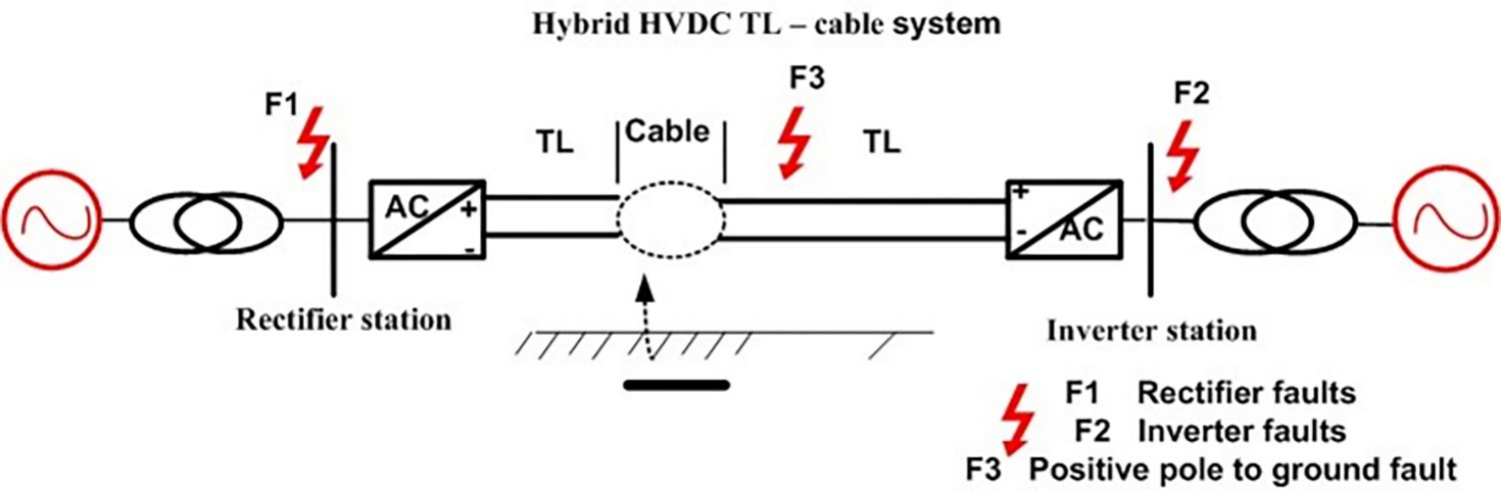
AC/DC fault location.

**Fig 18:**
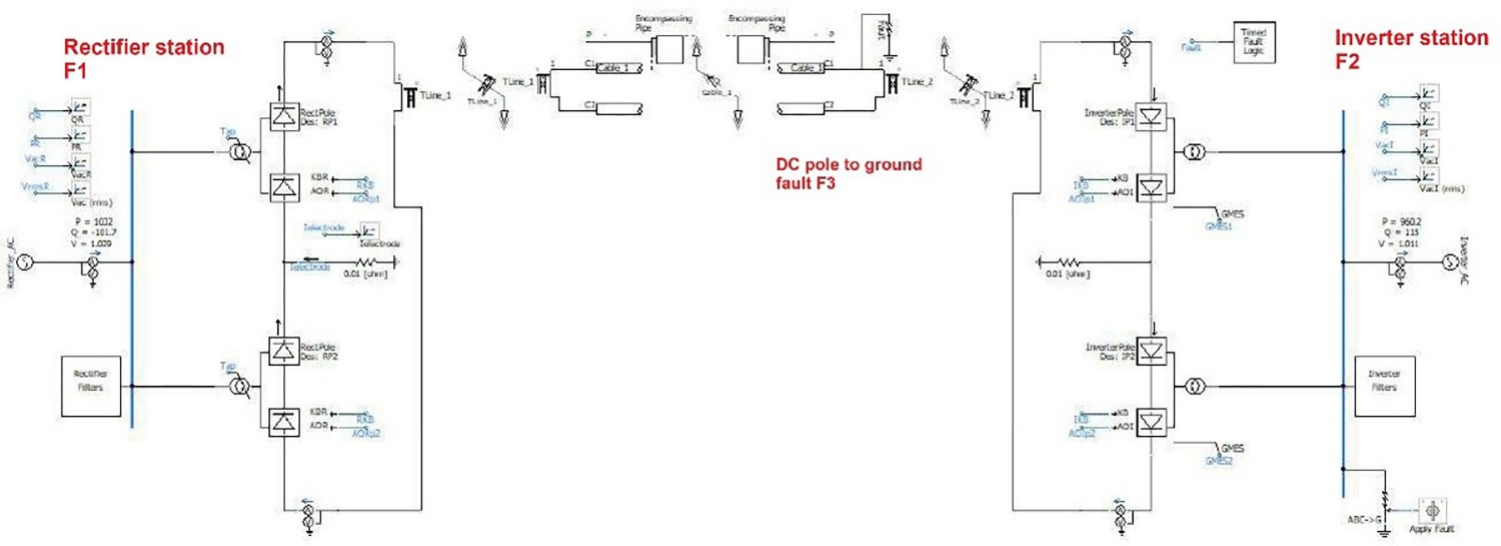
PSCAD HVDC fault model.

This section simulates fault analysis for AC/DC system faults such as Line- Line-line-ground (LLLG), Line-line-ground(LLG),line-ground(LG), and Dc fault Line -to- ground, as shown in Fig 17. The simulation duration is 2 seconds. Direct current and voltage are the output of the rectifier and inverter sides. The analysis of three scenarios was simulated as shown in Fig 18.

1. AC faults (LG, LLG, and LLLG)on the rectifier side (F1)
2. AC faults (LG, LLG, and LLLG) on the inverter side(F2)
3. Dc fault Line -to- ground (F3)

**Fig 19:**
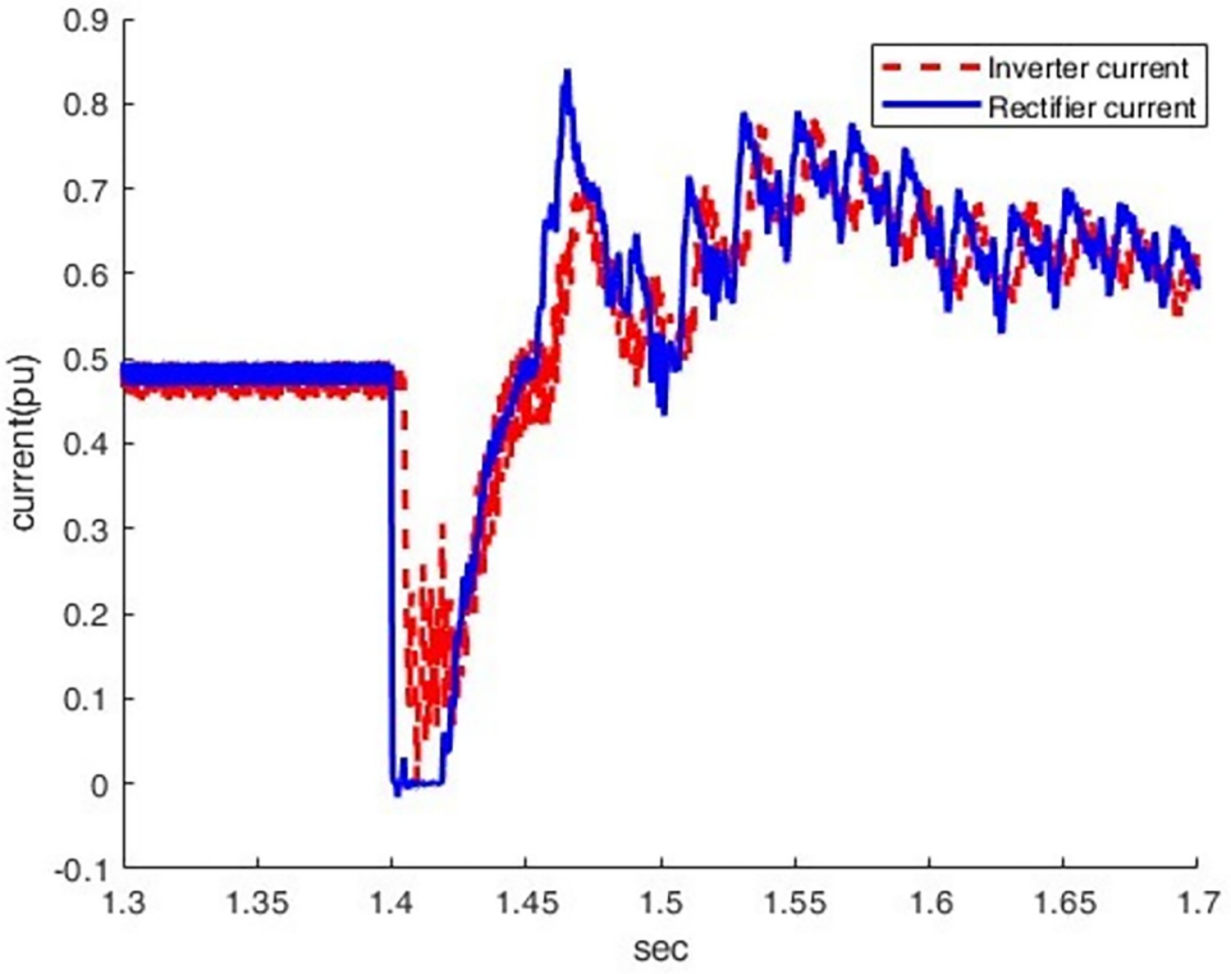
Current waveform at both sides.

**Fig 20:**
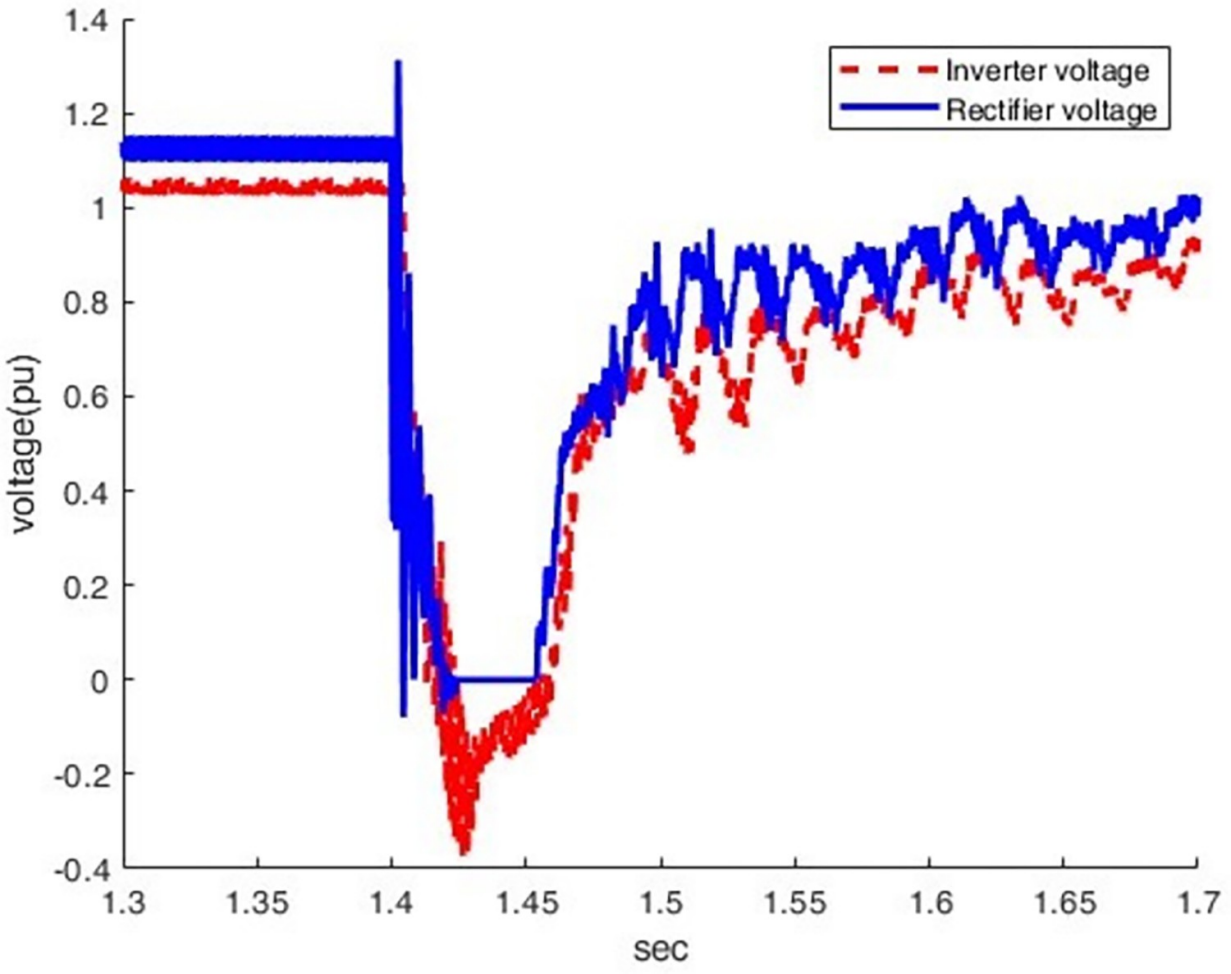
Voltage waveform at both sides.

*3*.*2*.*2*.*1*. *Scenario of AC faults on the rectifier side (F1)*. As shown in Figs 19 and 20, when LLLG fault is applied on the rectifier side, the rectifier voltage was increased to 1.3 pu as a temporary overvoltage. The overvoltage can be explained by the rapid release of energy stored in the submarine cable. This overvoltage is significantly influenced by submarine the cable capacitance, which discharges stored energy and results in the observed voltage spike [31]. The direct voltage of the rectifier station rose after 0.05s and then dropped to about the initial level. It also shows that if there were an LLLG fault at rectifier station F1, then the current would rise from 0.5 pu to 0.8 pu, resulting in an over current situation for the rectifier station.

**Fig 21:**
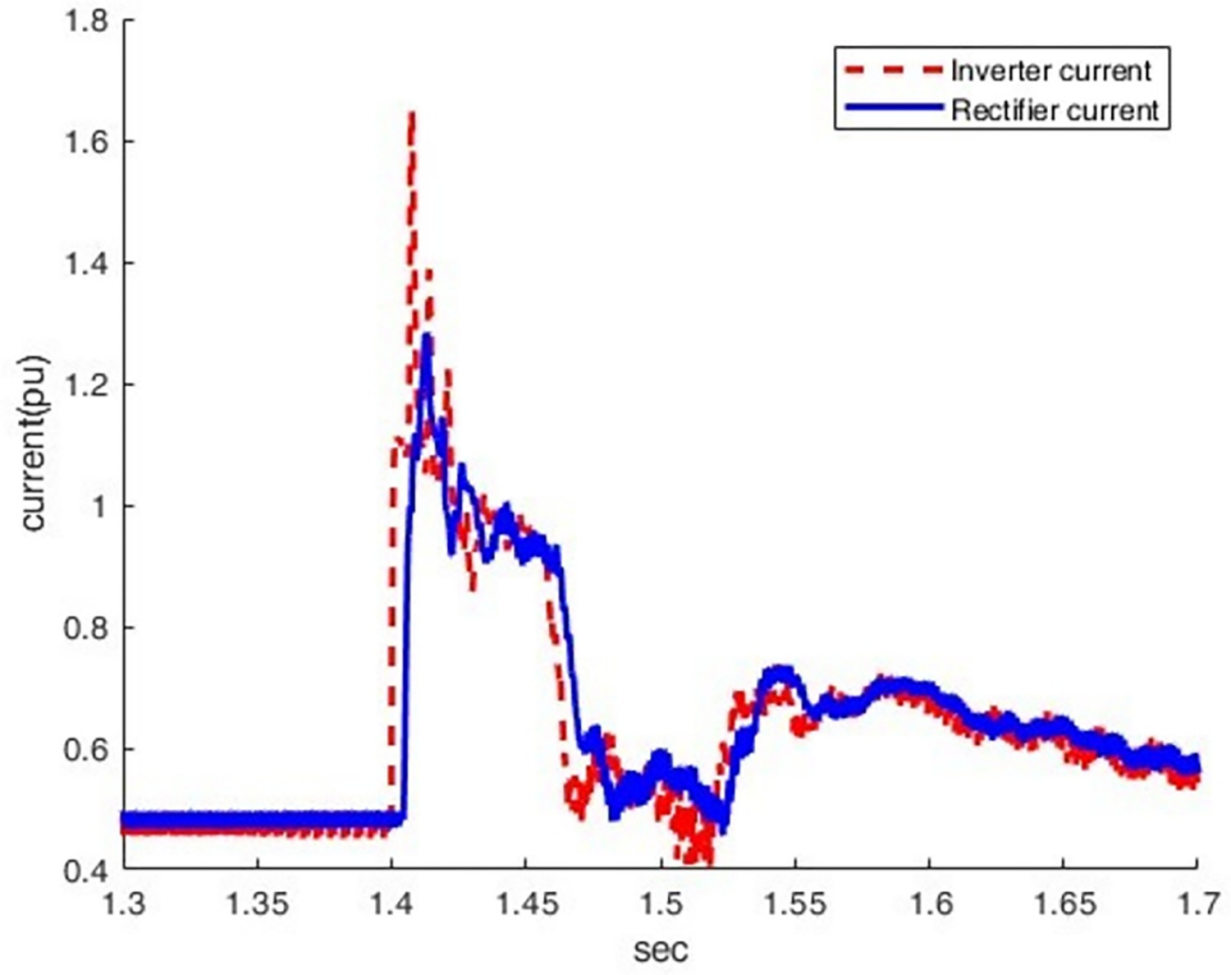
Current waveform at both sides.

**Fig 22:**
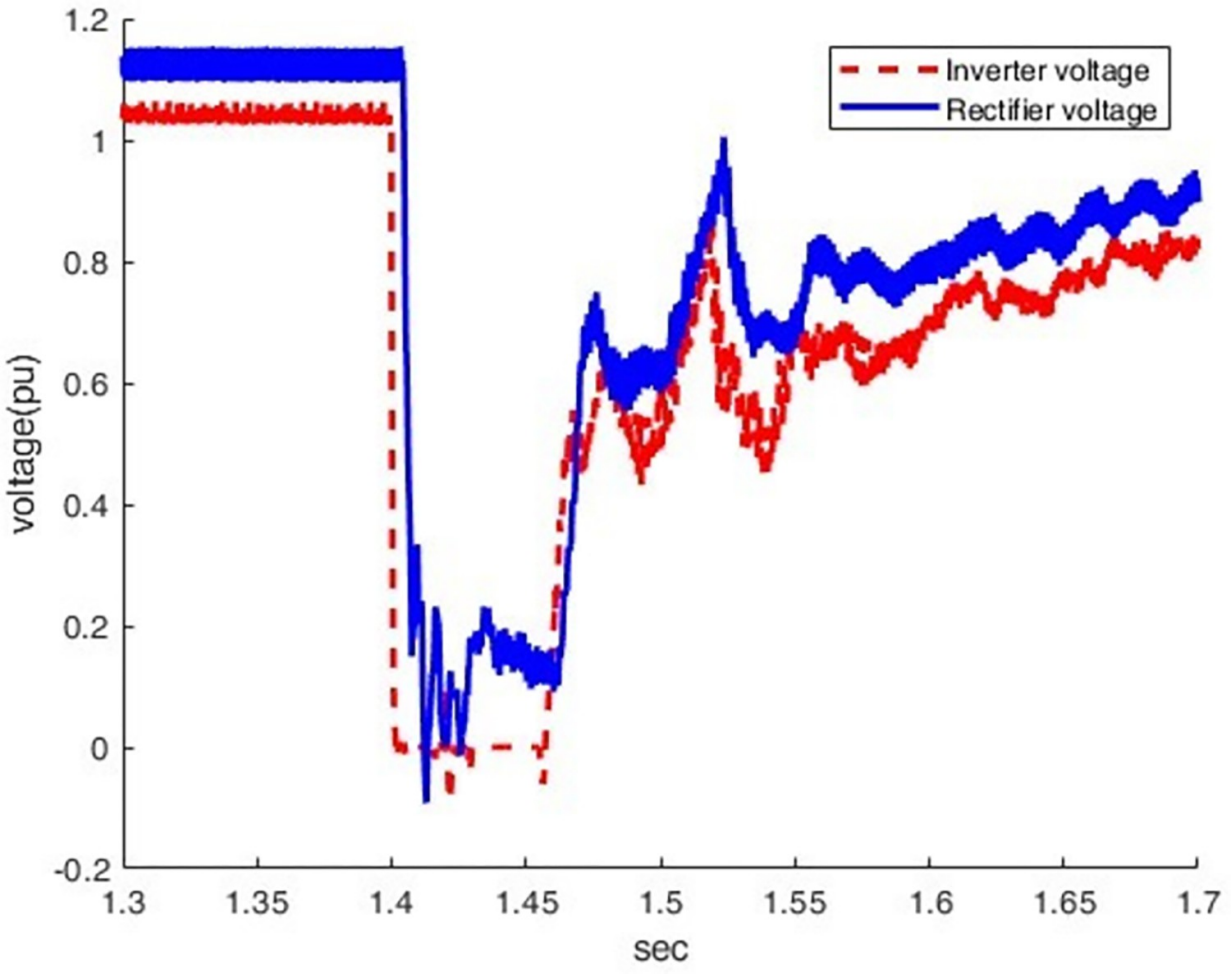
Voltage waveform at both sides.

*3*.*2*.*2*.*2*. *Scenario of three-phase grounding fault on the inverter side (F2)*. When LLLG fault occurs at the inverter station (F2), the current increases from 0.5 pu to 1.64pu, which causes a more severe overcurrent condition, as shown in Figs 21 and 22. At this moment, the direct voltage of the inverter station falls to zero pu while its direct current rapidly rises to three times the nominal value.

**Fig 23:**
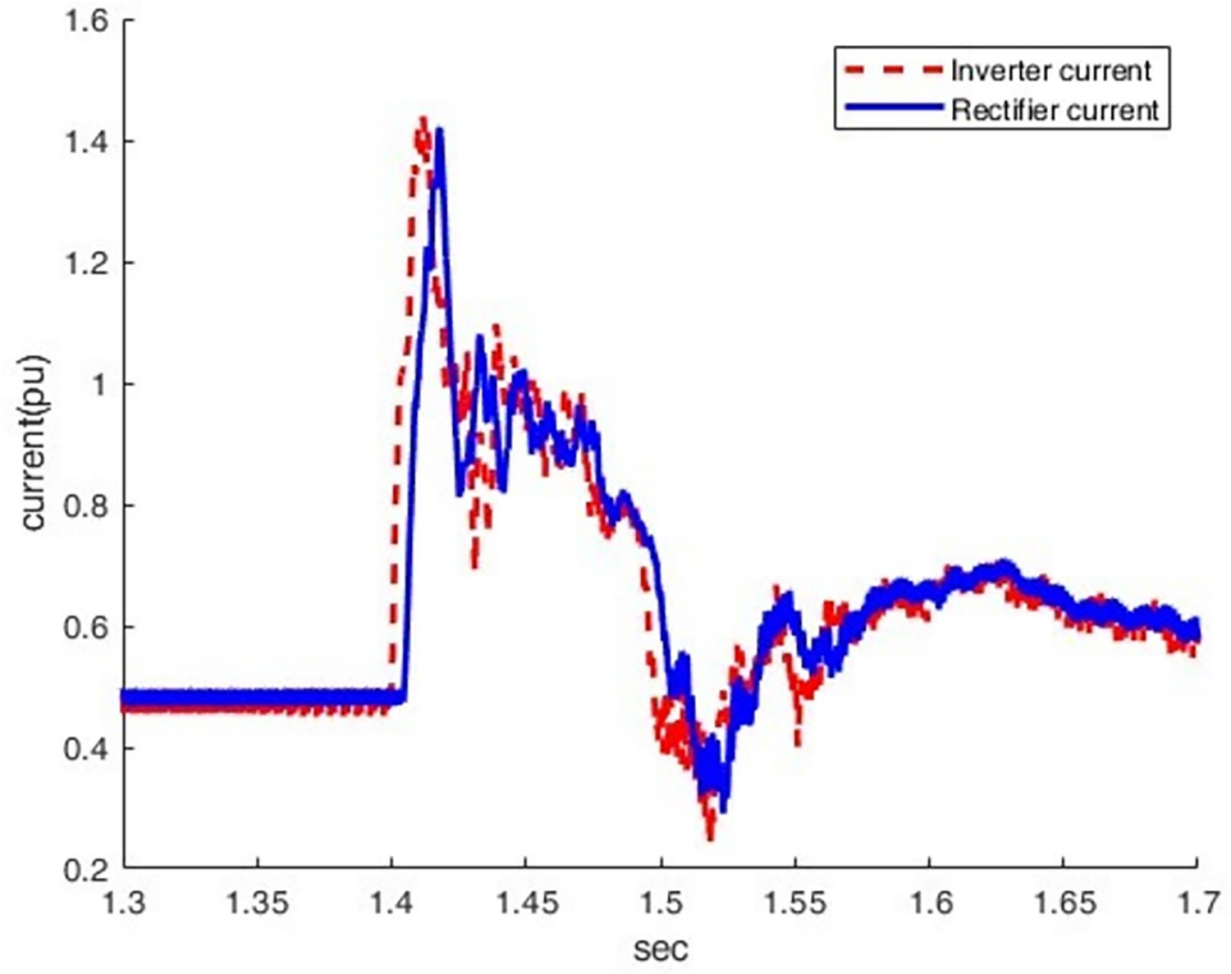
Current waveform at both sides.

**Fig 24:**
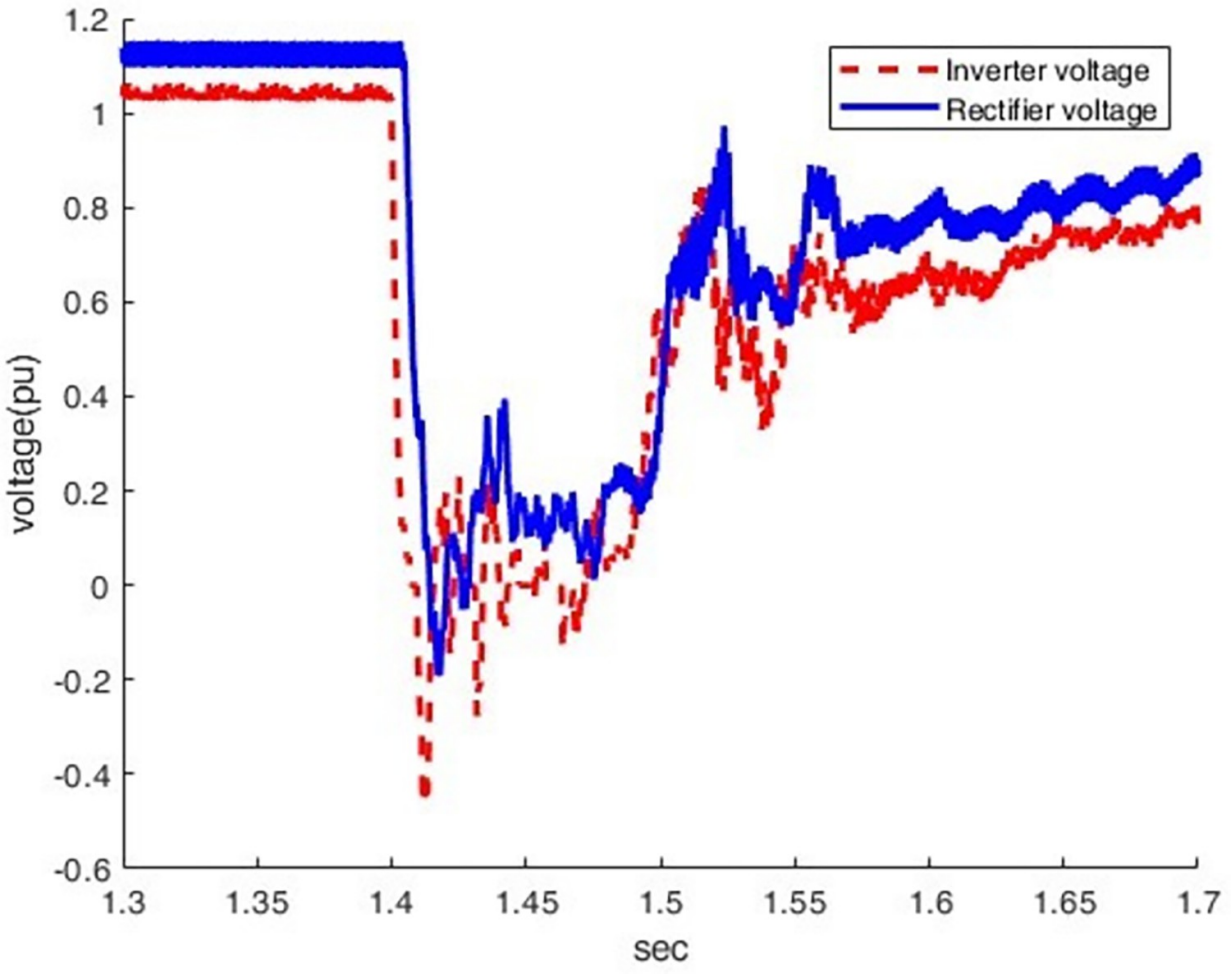
Voltage waveform at both sides.

When an LG fault occurs at the inverter station, the current increases from 0.5 pu to 1.43pu, which causes an overcurrent condition, as shown in Figs 23 and 24. At this moment, the direct voltage of the inverter station falls to zero pu.

**Fig 25:**
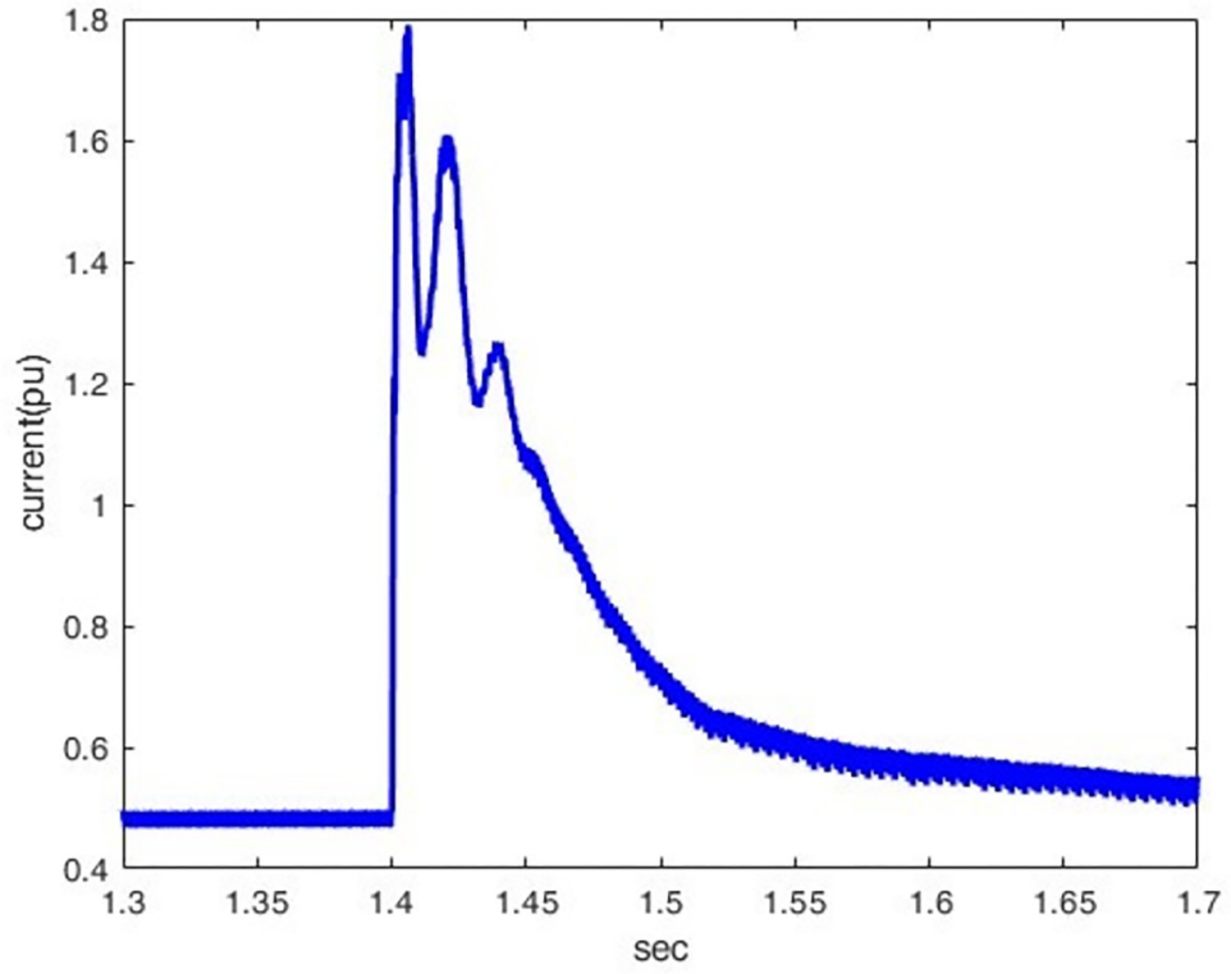
Current waveform under DC fault.

**Fig 26:**
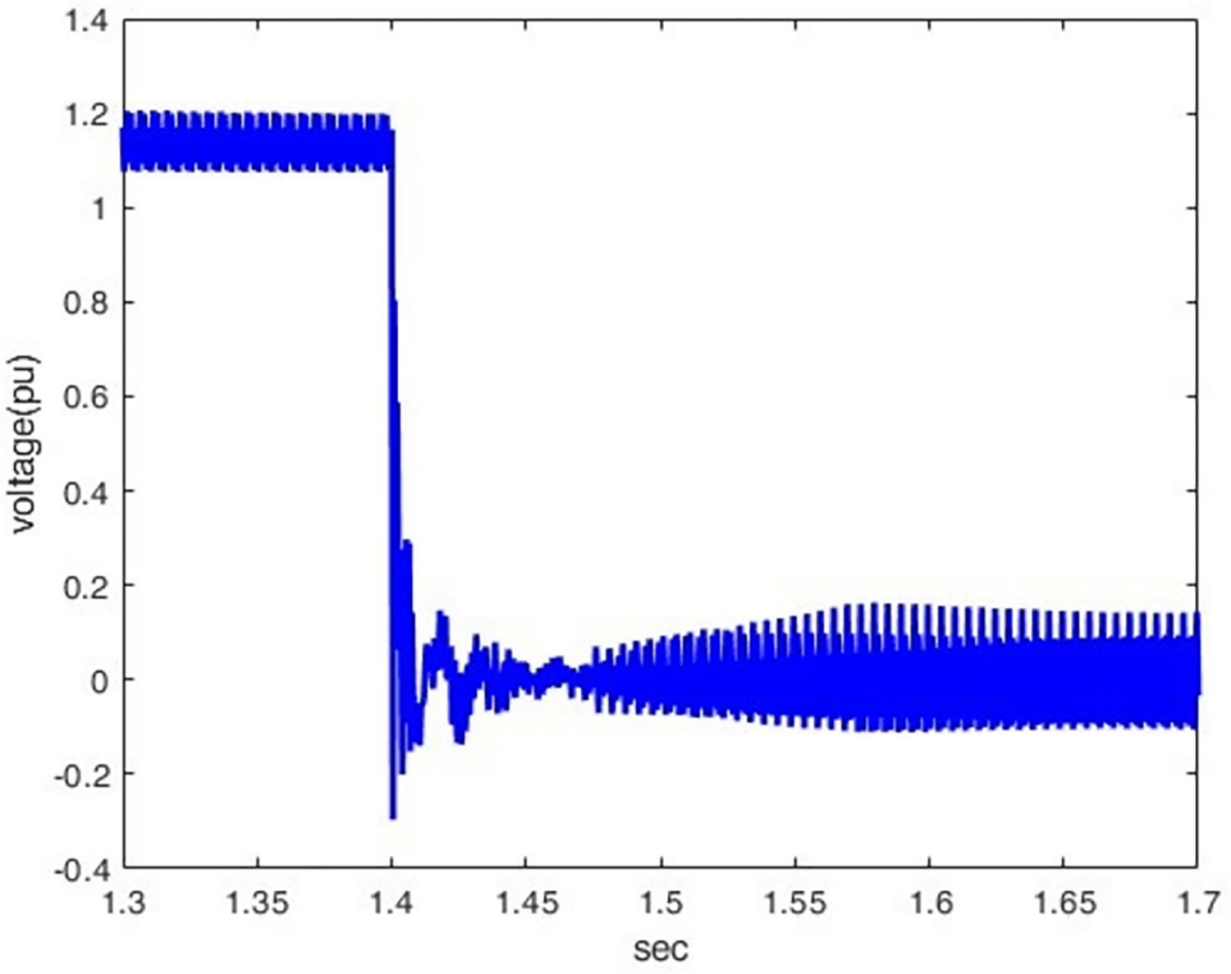
Voltagewaveform under DC fault.

*3*.*2*.*2*.*3*. *Scenario of Line -to- ground dc fault(F3)*. This introduces the line-to-ground DC fault(F3) and its impact on the rectifier station. As indicated by Figs 25 and 26, overcurrent increases from 0.5 p.u. to 1.78 p.u by making the DC fault the most sever condition for rectifier station. The voltage oscillates after a fault, indicating the difficulty of quickly restoring normal operation.

*3*.*2*.*2*.*4*. *Summary of faults results and discussion*. The analysis in Table 1 highlights that various fault types in the hybrid HVDC TL-cable have distinct effects on the rectifier and inverter sides. Particularly, they significantly impact current and voltage behaviour. It is noteworthy that the DC Pole-to-Ground fault stands out as the most severe fault scenario. This severity is due to the low impedance path created during the fault, resulting in a complete voltage collapse (0 pu) on both the rectifier and inverter sides. The fault also leads to significant disruptions in current, including sharp spikes or complete drops to zero. These characteristics underscore the heightened disruption associated with DC faults, making them more critical to manage compared to AC faults such as LG, LLG, and LLLG.

**Table 1:** Summary of faults results.

Rectifier Faults	Rectifier side		Inverter side	
	Current(pu)	Voltage(pu)	Current(pu)	Voltage(pu)
**LG**	1.64	1.22	1.23	0
**LLG**	1.08	1.223	1.157	0
**LLLG**	0.84	1.31	0.775	0
**DC Pole to ground**	1.78	0	0	0
**Inverter Faults**				
**LG**	1.41	0	1.43	0
**LLG**	1.31	0	1.63	0
**LLLG**	1.28	0	1.65	0

## 4. Conclusion

This paper proposes the PSCAD transient model of the ± 500 KV and 3000 MW grid connected from Badr City in Egypt to Madinah El Munawara in Saudi Arabia. The factors affecting the peak transient voltage at the sensitive points of the system and its decay time are analyzed. The work is performed using PSCAD. The following can be concluded:

1. As the peak lightning current increases, the resulting peak transient voltage rises, with the front and tail times maintained constant at 1.2/50 μs.
2. The wave front time is the most influential factor in determining peak transient overvoltage. As the front time decreases, the induced voltages at the rectifier station.
3. As the tail time increases, the induced voltages at the rectifier station also increase.
4. The overvoltage decreases when propagating from an HVDC TL to a submarine cable, so the overvoltage decreases at the rectifier side
5. This paper examines the performance of the Hybrid HVDC TL-cable configuration system during DC pole-ground faults and AC faults, focusing on both the rectifier and inverter stations. It observes that the DC pole-to-ground faults are more severe than other faults and lead to substantial overcurrent in the system, which may damage the rectifier station.
