# Expression of Concern: Assessment of the hazard risks on HVDC transmission networks due to lightning strikes and faults

**DOI:** 10.1371/journal.pone.0351353

**Published:** 2026-06-11

**Authors:** The PLOS One Editors

The *PLOS One* Editors issue this Expression of Concern for this article [1] because of concerns identified about the peer review process. Readers are advised to interpret the article [1] with caution.
